# Social Determinants of Association among Diabetes Mellitus, Visual Impairment and Hearing Loss in a Middle-Aged or Old Population: Artificial-Neural-Network Analysis of the Korean Longitudinal Study of Aging (2014–2016)

**DOI:** 10.3390/geriatrics4010030

**Published:** 2019-03-25

**Authors:** Kwang-Sig Lee, Kun Woo Park

**Affiliations:** 1 Center for Artificial Intelligence, Korea University College of Medicine, Seoul 02841, Korea; ecophy@hanmail.net; 2 Department of Neurology, Korea University College of Medicine, Seoul 02841, Korea

**Keywords:** association, diabetes mellitus, visual impairment, hearing loss, social determinant, old population

## Abstract

Background: This study introduces a new framework based on an artificial neural network (ANN) for testing whether social determinants are major determinants of association among diabetes mellitus, visual impairment and hearing loss in a middle-aged or old population. Methods: The data came from the Korean Longitudinal Study of Aging (2014–2016), with 6120 participants aged 45 years or more. The association was divided into eight categories: one category for having no disease, three categories for having one, three categories for having two and one category for having three. Variable importance, the effect of a variable on model performance, was used to evaluate the hypothesis based on whether family support, socioeconomic status and social activity in Y2014 are among the top 10 determinants of the association in the year 2016 (Y2016). Results: Based on variable importance from the ANN, brothers/sisters cohabiting (0.0167), voluntary activity (0.0148), income (0.0125), family activity (0.0125), parents alive (0.0121), leisure activity (0.0095) and meeting with friends (0.0092) in Y2014 are the top-10 determinants of comorbidity in Y2016. Conclusion: The findings of this study support the hypothesis, highlighting the importance of social determinants for the effective management of the comorbidities of the three diseases.

## 1. Introduction 

Diabetes mellitus, visual impairment and hearing loss are the leading causes of disease burden in the world [1,2,3,4,5,6]. The global prevalence of diabetes mellitus is expected to increase from 425 million in Year 2017 (Y2017 hereafter) to 693 million in Y2045 [1]. The number of people with visual impairments in the world rose from 191 million in Y1990 to 253 million in Y2015 [2]. The estimated prevalence of hearing loss in the world registered a rapid growth of 757% from 42 million in Y1985 to 360 million in Y2011 [3]. This global pattern is consistent with its local counterpart in South Korea (Korea hereafter). Diabetes mellitus was the fifth cause of death in the nation for Y2016 (i.e., 19.2 per 100,000) [4]. In terms of disability-adjusted life years per 100,000, otitis media (294), glaucoma (279) and diabetes mellitus (275) ranked seventh, eighth and ninth in the nation for Y2012, respectively [5]. 

Then, is there a strong association (or comorbidity) among the three diseases above, i.e., diabetes mellitus, visual impairment and hearing loss? What determines the association? Is it true that “social determinants of health” (or what the World Health Organization (WHO) calls “structural determinants of health such as socioeconomic capital, policy, status and value systems” [6]) are major determinants of this association? In fact, existing literature on the association among the three diseases is largely consistent with the notion of social determinants of health, which states that the incidence and progression of chronic diseases and their variation in society are not only affected directly by intermediate factors (such as biological conditions and health behaviors) but also mediated indirectly by structural/social determinants (including socioeconomic capital, policy, status and value systems) [6]: for instance, the damage of small blood cells [7,8], age [7,8,9,10], Body Mass Index (BMI) [7] and exercise [8] as the intermediate factors for the three diseases and marriage [8], self-rated health [8] and the quality of life [9,10] as their structural/social determinants. However, these studies center on a pair of the three diseases only. This line of research also suffers from paying attention to only a small set of factors for the association (excluding socioeconomic capital such as family support and social activity) and/or from having a cross-sectional research design (which cannot analyze a causal relationship between the association and its determinants). Indeed, new methods might be needed for the prediction of the association as a set of multiple dependent variables, given that making a prediction over a set of multiple dependent variables is much less accurate and effective than doing so for a single dependent variable. It might be a significant contribution to develop a framework (1) for identifying major determinants of association among multiple chronic diseases (e.g., all eight combinations of diabetes mellitus, visual impairment and hearing loss) and (2) for testing whether the association is very strong. It might be desirable for this framework to satisfy the following conditions as well: (3) including a large set of demographic, socioeconomic and health-related determinants for the association; (4) using nationally representative longitudinal data; and (5) introducing new approaches much more accurate and effective for making a prediction over a set of multiple dependent variables. 

In this context, this study develops a framework based on an artificial neural network (ANN) (1) to identify major determinants of association among all eight combinations of diabetes mellitus, visual impairment and hearing loss (as examples of chronic diseases in a middle-aged or old population) and (2) to test whether the association is very strong. The ANN, the central model of this study, has been known for its performance comparable or superior to those of traditional methods such as (multinomial) logistic regression and the random forest, regarding the prediction of chronic diseases [11,12,13,14]. Indeed, this study is characterized by (3) nationally representative longitudinal data for Koreans aged 45 years or more; (4) 30 demographic, socioeconomic and health-related determinants for the association; and (5) a new approach called “powerset methods”, which changes a “multi-label” classification design (with three dependent variables) to its “multi-class” counterpart (with eight categories of one dependent variable). In addition, this study might present useful implications, especially for nations similar with Korea, which is experiencing a rapid expansion of old populations and a sudden change in the family structure (e.g., an abrupt rise of the one-person family). This study adopts the WHO definition of “social determinants of health” as socioeconomic capital and status (education, income, occupation, family support and social activity), testing the following hypotheses from the literature and discussion above:

**Hypothesis** **1.** Social determinants are major determinants of association among diabetes mellitus, visual impairment and hearing loss in a middle-aged or old population.

**Hypothesis** **2.** The association among diabetes mellitus, visual impairment and hearing loss is very strong in a middle-aged or old population.

## 2. Methods 

### 2.1. Participants 

Data came from the Korean Longitudinal Study of Aging (KLoSA) in Y2014 and Y2016. The data were publicly available and de-identified. The KLoSA is designed to create nationally representative longitudinal data on Koreans aged 45 years or more, which help to trace their characteristics over time and to develop socioeconomic policies for these rapidly growing populations. This biennial survey involves a multistage stratified sampling based on geographical areas and housing types across the nation. It uses computer-assisted personal interviewing and covers a wide range of demographic, socioeconomic and health-related topics. The panels in the 1st, the 5th and the 6th waves for Y2006, Y2014 and Y2016 consisted of 10254, 8387 and 7893 subjects aged 45 or more, respectively. Among these 7893 subjects, 1773 subjects were excluded in this study, given that they lacked demographic, socioeconomic or health-related information. The final sample of this study consisted of 6120 subjects aged 53 or more (75% of whom were older than 60 as in Y2014). Despite the censoring of participants by the listwise deletion, there were no significant differences between the Y2016 sample and the analytic sample on the measures of interest. 

### 2.2. Measures 

Diabetes Mellitus, visual impairment and hearing loss in Y2014 and Y2016: The KLoSA question on diabetes mellitus in Y2014 and Y2016 was “Since the last survey, have you ever been diagnosed by a doctor as diabetes mellitus? 1. Yes. 5. No.” (C011) The inquiry on visual impairment (or hearing loss) in Y2014 and Y2016 was “Do you experience difficulty in daily activity because of visual impairment (or hearing loss)? 1. Yes. 5. No.” (C089 (or C092)). 

Disease–disease association in Y2016: The association among diabetes mellitus, visual impairment and hearing loss in Y2016 was divided into eight categories: (1) “0” for having no disease; (2) “1”, “2” and “3” for having diabetes mellitus only, visual impairment only and hearing loss only, respectively; (3) “4”, “5” and “6” for having diabetes mellitus and visual impairment, diabetes mellitus and hearing loss, and visual impairment and hearing loss, respectively; and “7” for having all three diseases. This approach, called “powerset methods”, changed a “multi-label” classification design (with three dependent variables) to its “multi-class” counterpart (with eight categories of one dependent variable). Here, “multi-label” means “many (dependent) variables” whereas “multi-class” means “many categories” of one dependent variable. 

Demographic, socioeconomic and health-related factors in Y2014: The following independent variables were also included in this study: (1) demographic factors, i.e., gender, age, marital status (married, separated, divorced, widowed and unmarried), the number of children alive, the number of brothers and sisters cohabiting, parents alive (father and mother, father, mother and none); (2) socioeconomic status including educational level (elementary school or below, junior high school, senior high school and college or above), personal income (normalized between 0 and 1), health insurance (Medicare and Medicaid) and economic activity (employed and unemployed); (3) social activity, i.e., monthly frequencies of meeting with friends, religious activity, friendship activity, leisure activity, family activity, voluntary activity and political activity; (4) health-related factors such as subjective health (very good, good, middle (neither good nor poor), poor and very poor), BMI, smoker (non, former and current), drinker (non, former and current) and drug/medication intake (yes and no); and (5) other determinants, i.e., religion (non, Protestant, Catholic, Buddhist, Won-Buddhist and other), residential type (apartment and other), region (big urban, small urban and rural), life satisfaction for economic status (0 to 100) and life satisfaction for overall life (0 to 100). 

### 2.3. Analysis 

Six popular machine learning methods were compared for the prediction of the association to check the reliability of the ANN: ANN, logistic regression, decision tree, naïve Bayes, random forest and support vector machine. A decision tree consists of (1) internal nodes (each meaning a test on an attribute (or independent variable)), (2) branches (each denoting an outcome of the test) and (3) terminal nodes (each representing a class label (or dependent variable)). A naïve Bayesian classifier is a predictor based on Bayes’ theorem. A random forest creates many training sets, trains many decision trees and makes a prediction with a majority vote (“bootstrap aggregation”). A support vector machine makes a prediction by maximizing a margin among hyperplanes separating the data. The ANN of this study includes one input layer, two hidden layers and one output layer with 91,800 neurons as data units in the input layer, 15 in each hidden layer and 8 in the output layer. The number of neurons in the input layer, 91,800, comes from the multiplication of 30 and 3060, which are the numbers of attributes and observations in the training set, respectively. Neurons in the input or previous hidden layer are combined with the weights in the next hidden or output layer (feedforward algorithm). Then, the weights in the output layer and its previous hidden layers are adjusted based on how much they contributed to the loss of the ANN, i.e., a gap between the actual and predicted class labels (backpropagation algorithm). Initially, the weights are set as small random numbers around 0 and the feedforward and backpropagation algorithms iterate until certain criteria are met for the accurate prediction of a class label [15]. 

The association among the three diseases with the eight categories in Y2016 served as the dependent variable of the models. Diabetes mellitus, visual impairment and hearing loss in Y2014 and the demographic, socioeconomic and health-related factors in Y2014 served as the independent variables of the models. The data on 6120 participants were divided into training and validation sets with a 50:50 ratio. The models were built (or trained) based on the training set with 3060 observations, and then, the models trained were validated based on the validation set with 3060 observations. Accuracy, a ratio of correct predictions among 3060 observations, was introduced as a criterion for validating the models trained. Variable importance from the ANN, an accuracy gap between a complete model and a model excluding a certain variable, was used for testing the two hypotheses of this study: (1) Hypothesis 1 was evaluated based on whether family support (e.g., parents/children alive), social activity (e.g., family/leisure activity) and socioeconomic status (e.g., education/income) in Y2014 are among the top-10 determinants of the association in Y2016, and (2) Hypothesis 2 was evaluated based on whether diabetes mellitus, visual impairment and hearing loss in Y2014 are among the top-5 determinants of the association in Y2016 (This can be considered one way of testing the correlation among the three diseases over time.). Finally, the greater “accuracy decrease” leads to the greater variable importance. This derivation is in a similar context but in the opposite direction of its random-forest counterpart: The variable importance from the random forest is a mean-impurity gap between a complete model and a model excluding a certain variable (The mean impurity, the degree of data being mixed at a node on average, is disproportional to accuracy.). The greater “mean-impurity increase” leads to a greater variable importance. Python 3.52 was employed for the analysis on December 2018. 

## 3. Results 

Table 1 shows the frequency tables for the participants’ disease–disease association and categorical attributes. Among the 6120 participants in Y2016, 1491 (24%) were diagnosed with at least one of the three diseases (diabetes mellitus, visual impairment and hearing loss) and 117 (2%) were characterized by a diagnosis of two or three of the diseases. Among the participants in Y2014, indeed, 1147 (19%), 202 (3%) and 104 (2%) were diagnosed with diabetes mellitus, visual impairment and hearing loss, respectively. Table 2 displays descriptive statistics for the participants’ continuous attributes. All (or 75%) of the participants in Y2014 were older than 53 (or 60). On average, the age of the participant was 68, the number of brothers and sisters cohabiting was 3, the monthly income was $1261 and the monthly frequency of family activity was 5. Based on Table 3, the ANN might be a reliable model with its accuracy (0.7507) being similar with those of logistic regression and the random forest (0.7507 and 0.7533, respectively). Indeed, variable importance from the ANN was derived by subtracting from the accuracy of the model with all variables (the ANN Full) (0.7507) the measure of the model excluding a certain variable (e.g., 0.7340 and 0.7382 for the ANN, excluding brothers/sisters cohabiting and income, respectively). 

**Figure 1:**
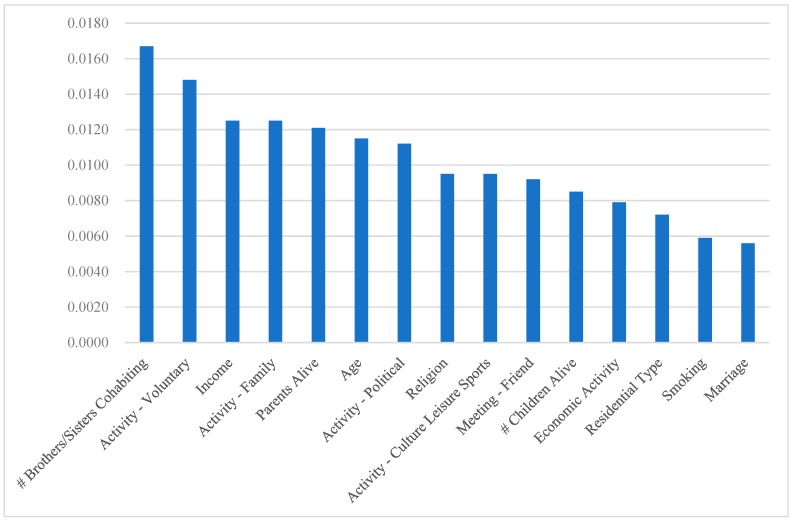
The variable importance from the Artificial Neural Network.

**Figure 2:**
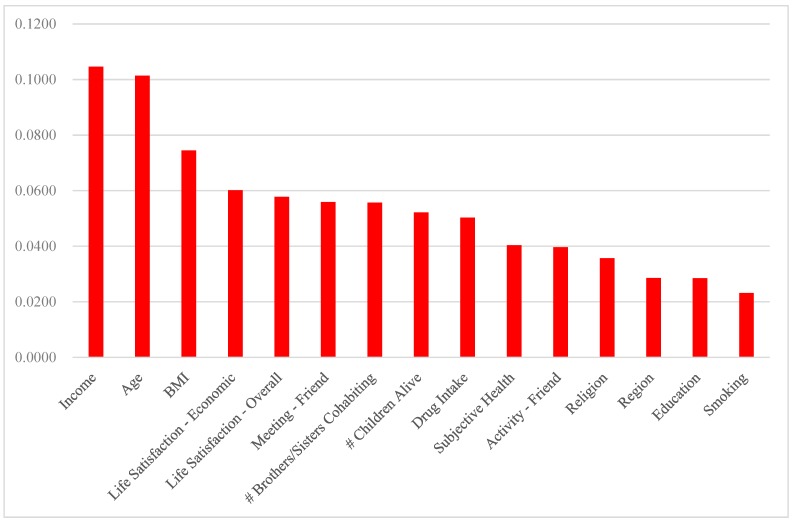
The variable importance from the Random Forest.

According to the variable importance from the ANN (Figure 1), brothers/sisters cohabiting (0.0167), voluntary activity (0.0148) and income (0.0125) in Y2014 are the top-3 determinants of the association in Y2016. Indeed, family activity (0.0125), parents alive (0.0121), leisure activity (0.0095) and meeting with friends (0.0092) in Y2014 are among the top-10 determinants of the association in Y2016 (These support Hypothesis 1.). The logistic regression results (Table S1, a supplementary table) provide useful information about the sign and magnitude for the effect of the major determinant on the association. For example, the odds of diabetes mellitus in Y2016 is 100 times as high for those with one unit of income (dollar) lower in Y2014 as for those with one unit of income (dollar) higher in Y2014 (This is also similar for hearing loss.). In addition, based on the variable importance from the random forest (Figure 2), income (0.1046), meeting with friends (0.0559), brothers/sisters cohabiting (0.0557) and children alive (0.0521) in Y2014 are among the top-10 determinants of the association in Y2016. The results of the ANN and the random forest both highlight the significance of promoting family support, social activity and socioeconomic status in disease control (According to both results, diabetes mellitus, visual impairment and hearing loss in Y2014 are out of the list for the top-10 determinants of the association in Y2016.). Finally, Figure S1 (a supplementary figure) shows the receiver-operating-characteristic (ROC) curve of the ANN over each of the 8 categories, the plot of the true positive rate (or sensitivity) vs. the false positive rate (or 1 − specificity). The area under the ROC curve (AUC) measures the power or usefulness of the model. Based on the measure, the ANN might be a powerful model: The AUC averaged over the 8 categories was 0.77. 

## 4. Discussion 

### 4.1. Summary of Findings 

The ANN might be reliable with its accuracy being similar with that of the logistic regression for the prediction of the association among diabetes mellitus, visual impairment and hearing loss in a middle-aged or old population. Based on the variable importance from the ANN, brothers/sisters cohabiting, voluntary activity and income in Y2014 are the top-3 determinants of the association in Y2016. Indeed, family activity, parents alive, leisure activity and meeting with friends in Y2014 are among the top-10 determinants of the association in Y2016. 

### 4.2. What is Already Known on the Topic 

There exists a positive linkage between a pair of diabetes mellitus, visual impairment and hearing loss through the damage of small blood vessels, which is affected by age, BMI, exercise, marriage, self-rated health and the quality of life. 

### 4.3. What This Study Adds 

This study uses an ANN for testing (1) whether social determinants are major determinants of association among diabetes mellitus, visual impairment and hearing loss in a middle-aged or old population and (2) whether the association among the three diseases is very strong in the middle-aged or old. Indeed, this study satisfies desirable conditions such as (3) nationally representative longitudinal data for Koreans aged 45 years or more; (4) 30 demographic, socioeconomic and health-related determinants for the association; and (5) a new approach called “powerset methods”, which changes a “multi-label” classification design (with three dependent variables) to its “multi-class” counterpart (with eight categories of one dependent variable). The results of this study do not support Hypothesis 2: diabetes mellitus, visual impairment and hearing loss in Y2014 are out of the list for the top-10 determinants of the association in Y2016 (This might indicate that the association among the three diseases might not be as strong as expected.). However, the findings of this study support Hypothesis 1, and the ANN puts more focus on various social activities (voluntary, family, political, leisure and friendship) than the random forest (friendship). As explained in the Methods Section, the feedforward and backpropagation algorithms with constant learning (i.e., continued updates of weights) iterate in the ANN until certain criteria meet for the accurate prediction of a class label. This unique process of the ANN might lead to its distinctive outcomes from other machine learning methods including the random forest. 

In addition, this study draws the following policy implications, given that family support (brothers/sisters cohabiting and parents alive), social activity (voluntary, family, leisure and friendship activities) and socioeconomic status (income) in Y2014 are found to be among the top-10 determinants of the disease–disease association in Y2016. Firstly, the promotion of family support and social activity among those aged 60 or more might be needed to prevent the comorbidities of diabetes mellitus, visual impairment and hearing loss in the population in Korea. As a matter of fact, family support and social activity among Koreans aged 60 or more was found to be still low, and economic burden was reported to be a major reason for the result [16,17,18]. In this context, the following strategies and actions might be imperative for encouraging family support and social activity to improve the health conditions of the old in the nation: strengthening family services for the old especially with chronic disease but no family either alive or nearby; expanding the system of vouchers and discount rates for social activity among old populations; creating more social institutions for the old, especially in rural areas; and bringing more variety into family support and social activities in these institutions, given that the demand for these areas is becoming more diverse across the age, sex, marital status, socioeconomic conditions and health-related characteristics of the old population [18,19,20]. 

Secondly, Korea’s social policy needs to be updated to improve the socioeconomic status of the old and to prevent the comorbidities of diabetes mellitus, visual impairment and hearing loss in the population in the nation. Korea has recently experienced an abrupt rise in the one-person family (i.e., from 6.9% to 27.2% during 1985–2015) [21] and a sudden advent of aged society: It took 115 years in France and 24 years in Japan but only 18 years in Korea for the proportion of elders (or those aging 65 years or older) to reach 14% (aged society) from 7% (aging society) [22]. Amidst these dramatic social transformations, however, the social protection for “unprepared” elders still remains much lower in Korea than in other advanced nations. The public social expenditure for the elderly constituted only 1.7% of the gross domestic product (GDP) in Korea for Y2007, only a quarter of the Organization for Economic Cooperation and Development (OECD) average for the same year [23]. The nation ranked 34th among 35 OECD members in terms of the GDP share of public social expenditure for Y2016 as well [24]. Several studies report the negative effects of socioeconomic status on disease-related morbidity or mortality as factors either independent or combined with nutrition, access to healthcare, etc. [25,26]. In this vein, a continued expansion of the social expenditure for the elderly might be a priority for Korea’s government policy to improve their socioeconomic status and to prevent comorbidities of the three diseases in the population.

### 4.4. Limitations of the Study

This study had some limitations. Firstly, this study used a weak form of the longitudinal design because of constraints on memory capacity: The association among diabetes mellitus, visual impairment and hearing loss with the eight categories in Y2016 (Wave 6) served as the dependent variable of the models, while the three diseases in Y2014 (Wave 5) and the demographic, socioeconomic and health-related factors in Y2014 (Wave 5) served as the independent variables of the models. Considering a stronger form of the longitudinal design, e.g., 2012–2016 (Waves 4, 5 and 6), brings too much burden on the computer. One solution for this issue is to employ a smaller number of determinants for the disease association; this might be an interesting topic for future research. Indeed, improving the memory capacity and employing the data in all six waves with a strong form of the longitudinal design is expected to improve the accuracy of the ANN much more. Secondly, this study did not consider the levels and durations of diabetes mellitus, visual impairment and hearing loss, which might be important factors for the association among the three diseases. Thirdly, this study used the KLoSA question on diabetes mellitus in Y2014 and Y2016, which might be misleading to some individuals with the diagnosis of diabetes mellitus prior to Y2014: They might consider the inquiry to be about a new diagnosis only, answering “No” to the inquiry (“Since the last survey, have you ever been diagnosed by a doctor as diabetes mellitus? 1. Yes. 5. No.” (C011)). Refining the question is expected to improve the quality of the research on the topic. Fourthly, expanding this study to other chronic diseases and other determinants of association such as health utility usage might add a great contribution to this line of research. Fifthly, this study did not consider the possible mediating effects among variables. Sixthly, a subgroup analysis, e.g., 45–54, 55–64, 65–74 and 75+ in age, might have offered more insight on the major determinants of the association among the three diseases. Finally, this study considered six popular machine learning methods (ANN, logistic regression, decision tree, naïve Bayes, random forest and support vector machine). Expanding this study to other machine learning methods such as the AdaBoost, the k-nearest neighbors, the convolutional neural network and the recurrent neural network might be a good topic for further research.

## 5. Conclusions

For preventing the comorbidities of diabetes mellitus, visual impairment and hearing loss in the middle-aged or old populations, the promotion of family support, social activity and socioeconomic status might be needed.

## Figures and Tables

**Table 1:** The frequency tables for participants’ disease–disease association and categorical attributes.

	Count	Percentage (%)
Association (in Y2016)		
N-N-N ^†^	4629	75.64
Y-N-N ^‡^	1157	18.91
N-Y-N	124	2.03
N-N-Y	93	1.52
Y-Y-N	65	1.06
Y-N-Y	40	0.65
N-Y-Y	9	0.15
Y-Y-Y	3	0.05
Education (in Y2014 Hereafter)		
Elementary or Below	2742	44.80
Junior High	1056	17.25
Senior High	1707	27.89
College or Above	615	10.05
Gender		
Male	2578	42.12
Female	3542	57.88
Marriage		
Married	4713	77.01
Separated	31	0.51
Divorced	127	2.08
Widowed	1209	19.75
Unmarried	40	0.65
Religion		
Non	3422	55.92
Protestant	1106	18.07
Catholic	384	6.27
Buddhist	1169	19.10
Won-Buddhist	13	0.21
Other	26	0.42
Residential Type		
Apartment	4093	66.88
Other	2027	33.12
Region		
Urban, Big	2508	40.98
Urban, Small	1964	32.09
Rural	1648	26.93
Parents Alive		
Father and Mother	267	4.36
Father	93	1.52
Mother	1029	16.81
None	4731	77.30
Health Insurance		
Medicare	5792	94.64
Medicaid	328	5.36
Economic Activity		
Employed	2395	39.13
Unemployed	3725	60.87
Subjective Health		
Very Good	68	1.11
Good	1618	26.44
Middle (Neither Good nor Poor)	2747	44.89
Poor	1387	22.66
Very Poor	300	4.90
Smoker		
Non	4245	69.36
Former	1080	17.65
Current	795	12.99
Drinker		
Non	3163	51.68
Former	921	15.05
Current	2036	33.27
Drug/Medicine Intake		
Yes	3856	63.01
No	2264	36.99
Diabetes Mellitus		
Yes	1147	18.74
No	4973	81.26
Visual Impairment		
Yes	202	3.30
No	5918	96.70
Hearing Loss		
Yes	104	1.70
No	6016	98.30

^†^ N-N-N for diabetes mellitus, no; visual impairment, no; and hearing loss, no. ^‡^ Y-N-N for diabetes mellitus, yes; visual impairment, no; hearing loss, no.

**Table 2:** Descriptive statistics for the participants’ continuous attributes.

	Mean	SD	Min	25%	50%	75%	Max
Age	67.90	9.59	53	60	67	75	105
Meeting with Friends	3.57	2.54	1	2	3	5	10
Activity: Religious	2.14	0.71	1	2	2	2	10
Activity: Friendship	4.04	1.46	1	4	4	4	10
Activity: Leisure	3.01	0.41	1	3	3	3	10
Activity: Family	5.03	0.62	1	5	5	5	10
Activity: Voluntary	4.00	0.15	1	4	4	4	10
Activity: Political	4.00	0.07	2	4	4	4	8
# Children Alive	2.94	1.43	0	2	3	4	9
# Brothers/Sisters Cohabiting	3.48	1.66	1	2	3	5	11
Income (Monthly, $)	1260.80	1642.64	0	300	684	1660	36,000
BMI	23.29	2.85	12	22	23	25	82
Life Satisfaction: Economic	53.68	19.77	0	40	60	70	100
Life Satisfaction: Overall	60.39	16.74	0	50	60	70	100

**Table 3:** The model performance and variable importance.

	Accuracy	RF-VI ^†^	ANN-VI ^‡^
Multinomial Logistic Regression	0.7507		
Decision Tree	0.6294		
Naive Bayes	0.1258		
Random Forest: 1000 Trees	0.7533		
Support Vector Machine	0.7542		
ANN Full	0.7507		
ANN Excluding # Brothers/Sisters Cohabiting	0.7340	0.0557	0.0167
ANN Excluding Activity: Voluntary	0.7359	0.0005	0.0148
ANN Excluding Income	0.7382	0.1046	0.0125
ANN Excluding Activity: Family	0.7382	0.0165	0.0125
ANN Excluding Parents Alive	0.7386	0.0145	0.0121
ANN Excluding Age	0.7392	0.1014	0.0115
ANN Excluding Activity: Political	0.7395	0.0002	0.0112
ANN Excluding Religion	0.7412	0.0357	0.0095
ANN Excluding Activity: Culture Leisure Sports	0.7412	0.0068	0.0095
ANN Excluding Meeting: Friend	0.7415	0.0559	0.0092
ANN Excluding # Children Alive	0.7422	0.0521	0.0085
ANN Excluding Economic Activity	0.7428	0.0151	0.0079
ANN Excluding Residential Type	0.7435	0.0161	0.0072
ANN Excluding Smoking	0.7448	0.0231	0.0059
ANN Excluding Marriage	0.7451	0.0193	0.0056
ANN Excluding Health Insurance	0.7454	0.0091	0.0053
ANN Excluding Gender	0.7461	0.0146	0.0046
ANN Excluding Hearing Loss	0.7461	0.0127	0.0046
ANN Excluding Region	0.7464	0.0286	0.0043
ANN Excluding Subjective Health	0.7474	0.0403	0.0033
ANN Excluding Activity: Friend	0.7474	0.0396	0.0033
ANN Excluding Diabetes Mellitus	0.7474	0.0205	0.0033
ANN Excluding Education	0.7477	0.0285	0.0030
ANN Excluding Activity: Religious	0.7480	0.0129	0.0027
ANN Excluding Life Satisfaction: Economic	0.7490	0.0601	0.0017
ANN Excluding Life Satisfaction: Overall	0.7490	0.0578	0.0017
ANN Excluding Drug/Medicine Intake	0.7497	0.0503	0.0010

^†^ The variable importance from the Random Forest (RF) for the excluded variable in the Artificial Neural Network (ANN). ^‡^ The variable importance from the ANN for the excluded variable in the ANN.

## Supplementary Materials

The following are available online at https://www.mdpi.com/2308-3417/4/1/30/s1. Table S1. The multinomial logistic regression results: the odds ratio for variable/association. Figure S1. The receiver-operating-characteristic curve from the Artificial Neural Network.
