# Supplementary material for: Social Determinants of Association among Diabetes Mellitus, Visual Impairment and Hearing Loss in a Middle-Aged or Old Population: Artificial-Neural-Network Analysis of the Korean Longitudinal Study of Aging (2014–2016)
Source: Geriatrics (Basel). 2019 Mar 25;4(1):30. doi: 10.3390/geriatrics4010030 (PMC6473411; doi:10.3390/geriatrics4010030)
Supplement: Supplementary file 1 [file geriatrics-04-00030-s001.zip › Supplementary Table-Figure.docx]

Table S1. Multinomial Logistic Regression Results: Odds Ratio for Variable/Association

| Association^†^ | YNN | NYN | NNY | YYN | YNY | NYY | YYY |
| --- | --- | --- | --- | --- | --- | --- | --- |
| Variable |  |  |  |  |  |  |  |
|  |  |  |  |  |  |  |  |
| Education |  |  |  |  |  |  |  |
| Elementary, Below |  |  |  |  |  |  |  |
| Junior High | 0.61 | 0.85 | 1.44 | 0.35 | 1.37 | 0.48 | 0.57 |
| Senior High | 1.39 | 0.94 | 0.39 | 0.76 | 4.03 | 1.43 | 0.85 |
| College, Above | 1.31 | *2.92 | 0.43 | 0.50 | 0.70 | 1.36 | 0.52 |
| Gender |  |  |  |  |  |  |  |
| Male |  |  |  |  |  |  |  |
| Female | 0.85 | 1.91 | 0.59 | 1.49 | 0.88 | 0.21 | 0.88 |
| Age | 0.98 | *1.04 | **1.12 | 1.00 | **1.13 | 1.03 | 1.01 |
| Marriage |  |  |  |  |  |  |  |
| Married |  |  |  |  |  |  |  |
| Separated | 0.16 | **0.07 | **0.07 | 0.09 | 0.32 | 0.89 | 1.01 |
| Divorced | 0.14 | 1.64 | 0.07 | 0.06 | 0.05 | 0.62 | 0.43 |
| Widowed | **2.31 | 1.07 | 1.19 | 2.04 | *3.87 | 1.74 | 1.07 |
| Unmarried | **0.02 | **0.04 | **0.08 | 0.01 | 0.01 | 0.88 | 0.29 |
| Religion |  |  |  |  |  |  |  |
| Non |  |  |  |  |  |  |  |
| Protestant | 0.88 | 1.24 | 1.26 | 1.89 | 0.38 | 0.43 | 2.07 |
| Catholic | 1.38 | 1.73 | 0.98 | 2.99 | 0.43 | 6.64 | 2.60 |
| Buddhist | 0.85 | **0.37 | 1.06 | 0.74 | *0.14 | 0.76 | 1.31 |
| Won-Buddhist | 0.12 | **0.01 | 1.29 | 0.33 | **0.04 | **1.30 | **0.34 |
| Other | 0.16 | **0.10 | **0.13 | 0.07 | 0.09 | *1.20 | 1.04 |
| Meeting with Friends | 0.96 | 0.91 | 1.01 | 0.93 | 0.89 | 1.23 | 1.19 |
| Activity - Religious | 1.03 | 1.00 | 0.75 | 1.19 | 1.28 | 0.48 | 0.81 |
| Activity - Friendship | 1.04 | 1.03 | 0.84 | 1.11 | 1.03 | 1.13 | 0.99 |
| Activity - Leisure | 0.78 | 0.97 | 0.64 | 0.52 | 1.77 | 0.60 | 0.85 |
| Activity - Family | 1.25 | 0.78 | 0.87 | 1.14 | 2.08 | 1.20 | 1.21 |
| Activity - Voluntary | 0.76 | 1.10 | 1.03 | 1.07 | 1.19 | 0.94 | 0.71 |
| Activity - Political | 1.82 | *0.08 | 0.19 | 3.78 | 0.02 | 1.55 | 0.98 |
| Residential Type |  |  |  |  |  |  |  |
| Apartment |  |  |  |  |  |  |  |
| Other | 0.46 | 0.47 | 1.27 | 1.15 | 2.14 | 1.11 | 0.10 |
| Region |  |  |  |  |  |  |  |
| Urban, Big |  |  |  |  |  |  |  |
| Urban, Small | *3.16 | 0.66 | 0.73 | 0.44 | 0.46 | 1.48 | 14.66 |
| Rural | **1.84 | 0.98 | 0.64 | 0.90 | 2.15 | 0.68 | 1.19 |
| # Children Alive | 1.03 | 1.05 | 0.87 | 0.88 | 1.25 | **2.24 | 1.01 |
| # Brothers/Sisters Cohabiting | *0.86 | **1.20 | *1.21 | 1.00 | 0.84 | 0.78 | 0.95 |
| Parents Alive |  |  |  |  |  |  |  |
| Father & Mother |  |  |  |  |  |  |  |
| Father | 2.42 | 0.24 | 1.09 | 0.07 | 2.36 | 1.10 | 0.68 |
| Mother | 2.48 | 14.67 | 0.23 | 0.64 | 0.97 | 0.70 | 1.14 |
| None | 2.77 | 15.28 | 0.21 | 0.75 | 2.24 | 0.45 | 0.93 |
| Health Insurance |  |  |  |  |  |  |  |
| Medicare |  |  |  |  |  |  |  |
| Medicaid | 1.07 | **2.96 | 1.35 | 0.43 | 3.12 | 0.05 | 0.61 |
| Economic Activity |  |  |  |  |  |  |  |
| Employed |  |  |  |  |  |  |  |
| Unemployed | 0.91 | 1.31 | 1.44 | 1.40 | 0.78 | 0.79 | 1.04 |
| Income (Monthly, $) | **0.01 | 41.59 | **0.02 | 339.94 | 0.30 | 1.10 | **1.74 |
| Subjective Health |  |  |  |  |  |  |  |
| Very Good |  |  |  |  |  |  |  |
| Good | 2.71 | **0.33 | 1.65 | 3.18 | 1.93 | 1.31 | 1.13 |
| Middle | 3.20 | 1.58 | **2.43 | 3.17 | 0.72 | 2.13 | 1.28 |
| Poor | 1.44 | **2.38 | **2.21 | 1.86 | 0.78 | 1.07 | 1.10 |
| Very Poor | *4.63 | **11.30 | *2.62 | *5.10 | 4.36 | 0.31 | 0.55 |
| BMI | **1.12 | 0.92 | 0.99 | 0.97 | **1.25 | 1.04 | 1.04 |
| Smoker |  |  |  |  |  |  |  |
| Non |  |  |  |  |  |  |  |
| Former | 1.26 | 0.99 | 0.85 | 1.09 | 2.17 | 0.15 | 0.84 |
| Current | 0.79 | 0.50 | 1.54 | 0.39 | 0.11 | 0.25 | 0.82 |
| Drinker |  |  |  |  |  |  |  |
| Non |  |  |  |  |  |  |  |
| Former | *1.93 | 1.60 | 1.22 | **5.89 | 1.96 | 2.02 | 1.60 |
| Current | 1.08 | 1.75 | 1.00 | 1.15 | 1.27 | 0.42 | 0.81 |
| Life Satisfaction-Economic | **0.98 | 0.99 | 1.00 | 0.98 | 1.00 | 0.97 | 0.99 |
| Life Satisfaction-Overall | 1.00 | *1.02 | **1.03 | 0.98 | 1.00 | 0.97 | 1.02 |
| Drug Intake |  |  |  |  |  |  |  |
| Yes |  |  |  |  |  |  |  |
| No | 1.31 | **0.57 | 1.06 | 0.44 | 0.45 | 2.59 | 1.09 |
| Diabetes Mellitus |  |  |  |  |  |  |  |
| Yes |  |  |  |  |  |  |  |
| No | **0.01 | 0.15 | 0.45 | **0.01 | **0.01 | **0.01 | **0.01 |
| Visual Impairment |  |  |  |  |  |  |  |
| Yes |  |  |  |  |  |  |  |
| No | 2.11 | **0.15 | 0.51 | 0.30 | 2.41 | *0.08 | 0.34 |
| Hearing Impairment |  |  |  |  |  |  |  |
| Yes |  |  |  |  |  |  |  |
| No | **4.36 | **127.32 | **0.19 | 1.41 | **0.19 | 1.74 | 0.30 |
|  |  |  |  |  |  |  |  |

| * | p-value < 0.10 | | | |
| --- | --- | --- | --- | --- |
| ** | p-value < 0.05 | | | |
| † | YNN for | Diabetes Milieus Yes | Visual Impairment No | Hearing Impairment No |
|  | YYY for | Diabetes Milieus Yes | Visual Impairment Yes | Hearing Impairment Yes |
|  | NNN for | Reference Class |  |  |

Figure S1. Receiver-Operating-Characteristic Curve from the Artificial Neural Network


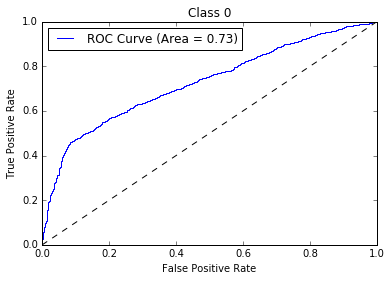

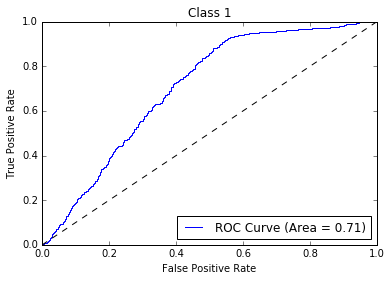

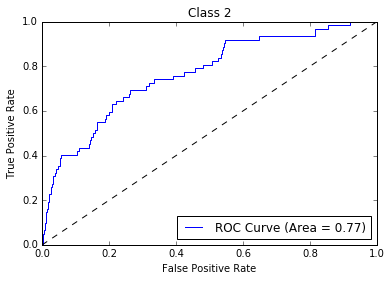


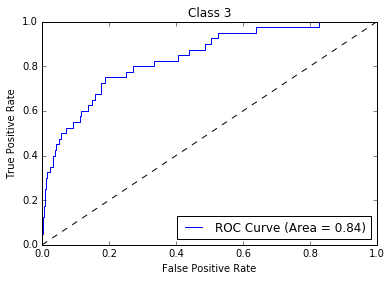

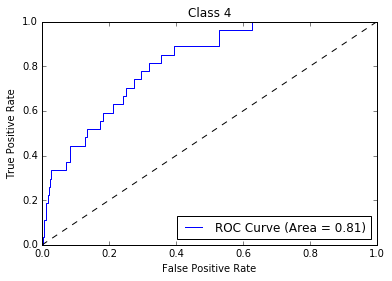

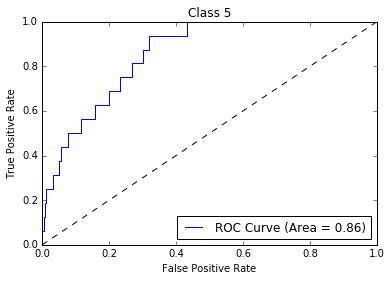


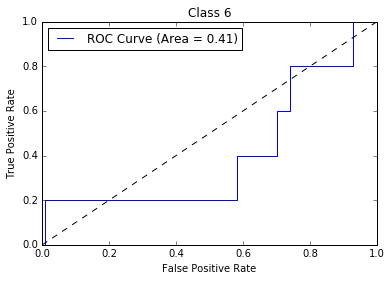

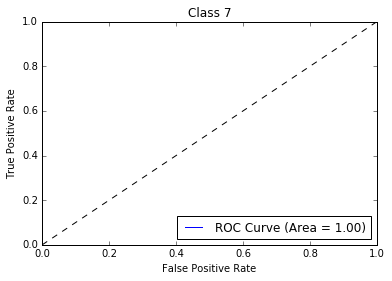


Note:

|  | Class 0 for | Diabetes Mellitus No | Visual Impairment No | Hearing Loss No |
| --- | --- | --- | --- | --- |
|  | Class 1 for | Diabetes Mellitus Yes | Visual Impairment No | Hearing Loss No |
|  | Class 2 for | Diabetes Mellitus No | Visual Impairment Yes | Hearing Loss No |
|  | Class 3 for | Diabetes Mellitus No | Visual Impairment No | Hearing Loss Yes |
|  | Class 4 for | Diabetes Mellitus Yes | Visual Impairment Yes | Hearing Loss No |
|  | Class 5 for | Diabetes Mellitus Yes | Visual Impairment No | Hearing Loss Yes |
|  | Class 6 for | Diabetes Mellitus No | Visual Impairment Yes | Hearing Loss Yes |
|  | Class 7 for | Diabetes Mellitus Yes | Visual Impairment Yes | Hearing Loss Yes |
